# Tensile Deformation and Fracture Behaviors of a Nickel-Based Superalloy via In Situ Digital Image Correlation and Synchrotron Radiation X-ray Tomography

**DOI:** 10.3390/ma12152461

**Published:** 2019-08-02

**Authors:** Qiang Zhu, Gang Chen, Chuanjie Wang, Heyong Qin, Peng Zhang

**Affiliations:** 1 Key Laboratory of Micro-systems and Micro-structures Manufacturing of Ministry of Education, Harbin Institute of Technology, Harbin 150080, China; 2 School of Materials Science and Engineering, Harbin Institute of Technology at Weihai, Weihai 264209, China; 3 Central Iron & Steel Research Institute, Beijing 100081, China

**Keywords:** superalloy, digital image correlation, synchrotron radiation, strain evolution, void defect, fracture mechanism

## Abstract

Nickel-based superalloys have become key materials for turbine disks and other aerospace components due to their excellent mechanical properties at high temperatures. Mechanical properties of nickel-based superalloys are closely related to their microstructures. Various heat treatment processes were conducted to obtain the desired microstructures of a nickel-based superalloy in this study. The effect of the initial microstructures on the tensile deformation and fracture behaviors was investigated via in situ digital image correlation (DIC) and synchrotron radiation X-ray tomography (SRXT). The results showed that the size and volume fraction of γ″ and γ′ phases increased with the aging time. The yield strength and the ultimate tensile strength increased due to the precipitation strengthening at the expense of ductility. The surface strain analysis showed severely inhomogeneous deformation. The local strains at the edge of specimens were corresponded to higher void densities. The fracture of carbides occurred owing to the stress concentration, which was caused by the dislocation accumulation. The fracture mode was dimple coalescence ductile fracture.

## 1. Introduction

Nickel-based superalloys have been extensively applied in aerospace and nuclear industries due to their marvelous mechanical properties which include high temperature strength, decent oxidation and corrosion resistance, outstanding fatigue property, and fracture toughness [1,2,3,4]. The excellent mechanical properties of nickel-based superalloys mainly depend on the precipitation strengthening by various precipitates, solution strengthening by plenty of alloying elements, and the interface microstructure of the coherent phase [5,6,7]. Generally, the complex precipitates in Inconel 718 (also known as GH4169) play an important role in mechanical properties, which include a primary strengthening phase γ″ (Ni_3_Nb) with an ordered body-centered tetragonal (D0_22_) structure, an assistant strengthening phase γ′ (Ni_3_Al) with an ordered face-centered cubic (L1_2_) structure, an equilibrium phase δ (Ni_3_Nb) with an orthorhombic (D0_a_) structure, and a fine carbide particle.

Many studies were concentrated on the effect of microstructures on the deformation and fracture behaviors of Inconel 718 [8,9,10]. Moussaoui et al. [11] and Wang et al. [12] found that the precipitation of γ″ and γ′ phases in the matrix γ phase enhanced the mechanical characteristics. Lin et al. [13] and Ning et al. [14] proposed that the mechanical properties were closely related to the δ phase and the occurrence of the δ phase deteriorated the ductility. Chang et al. [15] showed that the grain boundary strength decreased owing to the occurrence of brittle carbide and δ phase. Zhang et al. [16] found that the fracture of carbides (NbC) and the interfacial debonding of carbide/matrix γ phase resulted in the formation of void defects. Therefore, the influence of various precipitates on the mechanical properties and fracture behavior of Inconel 718 is quite complex.

Another critical issue is the formation of void defects, which is particularly important for key components that require high strength and fatigue resistance [17]. The post-mortem examination of deformed and fractured microstructures was widely used for the research approaches of defect development, which could not realize 3D visualization or obtain statistically meaningful quantitative data [18,19,20,21,22]. The formation of void defects is closely related to strain evolution during plastic deformation. Digital image correlation (DIC) is a non-contact measurement technique in the field of strain measurement. The DIC technique converts the color value of the points in the image into the corresponding digital signal by using the correlation between displacement and image, which realizes the dynamic recording of the position of the points during the whole deformation. By comparing and calculating the position of the points in the deformed and the undeformed material pictures, the dynamic observation of the strain is realized. As a non-destructive volume characterization technique with high strength, high collimation, and high polarization, synchrotron radiation X-ray tomography (SRXT) can 3D characterize internal material features. Therefore, the coupling of DIC and SRXT techniques can realize 2D and 3D direct visualization research of strain evolution and void defects. This has an important role in the study of the fracture behavior of nickel-based superalloys.

The study of the microstructure evolution of superalloys is important to the understanding of the relationship between microstructures and properties. The microstructure evolution of Inconel 718 was investigated via various heat treatments in this study. The effects of γ″ and γ′ phases with various sizes and volume fractions on the tensile deformation and fracture behavior were investigated via DIC and SRXT techniques.

## 2. Materials and Methods

**Figure 1:**
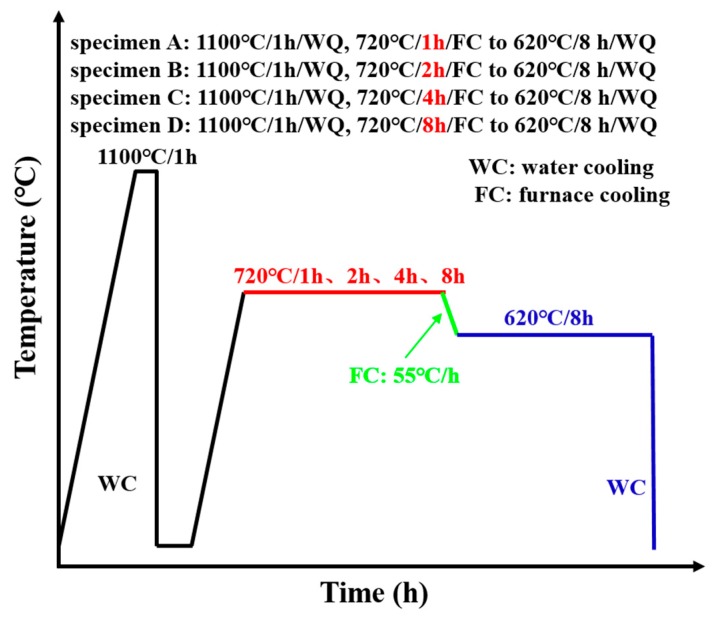
Specific details of the heat treatment process.

A commercial Inconel 718 sheet with a thickness of 200 μm was employed in the present study. The actual chemical composition was as follows (in wt%): Ni 52.80, Cr 18.73, Nb 5.24, Mo 3.02, Al 0.52, Ti 0.95, C 0.026, Co 0.03, Fe the rest. The tensile specimens for DIC were machined to 5 mm in gauge width and 12 mm in gauge length using electrical discharge machining (EDM). The tensile specimens for SRXT were machined to 300 μm in gauge width and 3 mm in gauge length using EDM. After the tensile specimen machining, they were cleaned with an acetone solution in an ultrasonic cleaner to remove the surface cutting fluid. A 2000 grit SiC paper was used to polish the edge of the specimen to reduce the impact of EDM on the performance of the specimens. Then the tensile specimens were cleaned with an alcohol solution in an ultrasonic cleaner. A vacuum heat treatment method was adopted to prevent specimen oxidation. A reasonable heat treatment process was very important for obtaining the desired microstructures of nickel-based superalloys. The microstructure evolution of nickel-based superalloys under different heat treatment conditions was studied [23,24]. Generally, the precipitation temperature range of γ″ phase in Inconel 718 superalloy was 600–900 °C, and the precipitation temperature range of δ phase was 750–1020 °C [13,25]. To comprehensively investigate the influences of initial microstructures on the plastic deformation mechanism and fracture characteristics, the tensile specimens were first solution-treated at 1100 °C with water quenching (WQ) and then aging-treated at 720 °C with furnace cooling (FC) at 55 °C/h to 620 °C/8 h with WQ. Four kinds of heat treatment procedures were carried out so as to precipitate only γ′/γ″ phases with various sizes and volume fractions before uniaxial tensile tests. The specific details of the heat treatment process are shown in Figure 1. The specimens were denoted as specimen A, specimen B, specimen C, and specimen D.

**Figure 2:**
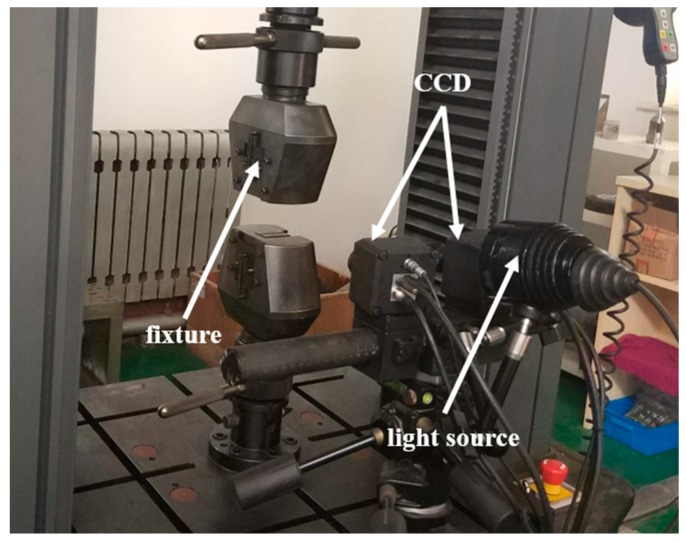
Device schematic of the digital image correlation (DIC) technique.

After full heat treatments, the uniaxial tensile tests with a low strain rate of 0.001 s^−1^ were performed on a universal testing machine (Instron5967, INSTRON, Boston, MA, USA) at room temperature. The software automatically plotted the force–displacement curve during the tensile test. The strain rate was within the appropriate window to report corresponding data such as yield strength and tensile strength. Since the gauge length of the tensile specimen was too small, the strain gauge was not used. To measure yield strength accurately, MATLAB software (MathWorks, Natick, MA, USA) was used to fit the elastic region in the engineering stress–engineering strain curve. Therefore, these values were only good for comparison within this test. To reduce experimental error, four identical specimens were tested under each condition. To obtain accurate strain field, the image, which was acquired during the tensile test, should contain enough speckle spraying. The deformation information for strain development could be calculated by the DIC system. The device schematic of the DIC technique is shown in Figure 2. To measure strain development, the specimen surface was first treated with speckle spraying, which covered the specimen surface with a layer of white paint and then evenly painted it with carbon powder. Speckle spraying was employed to obtain a clear and uniform black spot on the white surface, so that it was convenient to track the position of the point and calculate the displacement and strain. Many initial undeformed to final fractured TIFF images were taken at the gauge length of each tensile specimen with the exposure time of 250 ms. The first step for strain analysis calculation was the mesh division using an XTDIC-3D (XJTOP, Suzhou, China) digital speckle strain measurement and analysis system. Subsequently, the DIC analysis program captured and tracked the coordinates of the node position. Following this, the position coordinates of each node at each stage during the deformation process were obtained. Finally, the displacement and strain were calculated.

**Figure 3:**
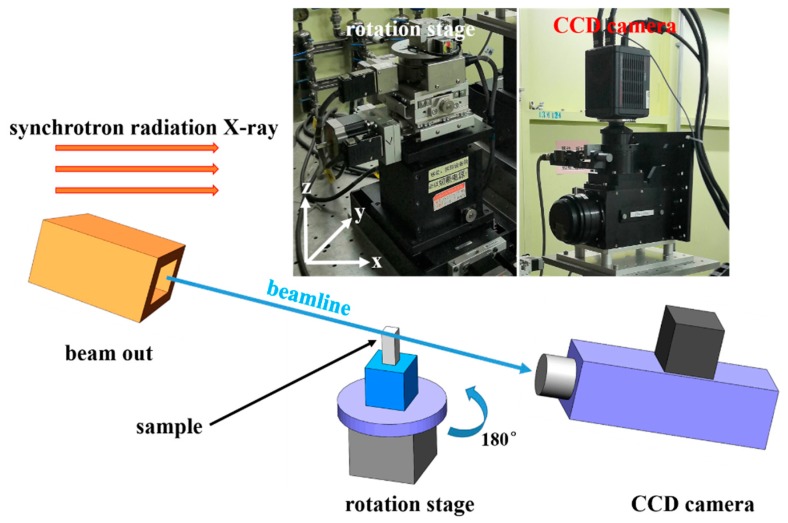
Device schematic of the synchrotron radiation X-ray tomography (SRXT) technique.

The spatial distribution of void defects was characterized at the BL13W1 beamline of the Shanghai Synchrotron Radiation Facility (SSRF) by SRXT (SSRF, Shanghai, China). Figure 3 shows the device schematic of the SRXT technique. One thousand images were obtained with a beam energy of 34 keV, a spatial resolution of 0.65 μm, and an exposure time of 250 ms for each projection. The 2D phase recovery, slice reconstruction, and artifact removal of raw images were carried out by the Fourier transform method in PITRE software (SSRF, Shanghai, China). The 3D volume reconstruction of the deformed tensile specimen and void defects was performed in Avizo 9.0 software (Thermo Fisher Scientific, Boston, MA, USA) with a Gaussian filter and a median filter treatment. Following this, the threshold values for the void defects, fractured tensile specimen, and the background were adjusted in segmentation module. Finally, the quantitative statistics and analysis of the void characteristics were performed.

For microstructure observation, the heat-treated specimens were conventionally ground and mechanically polished, followed by electrolytic polishing in a mixed solution of 20 mL HClO_4_ + 180 mL C_2_H_5_OH at 25 V. The microstructures were characterized via scanning electron microscopy (SEM) (ZEISS, Oberkochen, Germany) equipped with an electron backscattered diffraction (EBSD, EDAX, Mahwah, NJ, USA) system. A scanning step size of 0.5 μm and a binning of 4 × 4 were used for EBSD characterization. Areas of approximately 500 × 400 μm were observed on the rolling direction-transverse direction (RD-TD) plane. TSL OIM Analysis 7 software (EDAX, Mahwah, NJ, USA) was employed for the EBSD analysis. To reveal the details of γ′ and γ″ phases, transmission electron microscope (TEM) observations were performed on a Tecnai G2 F30 (FEI, Hillsboro, OR, USA). Transverse slices were mechanically ground to a thickness of approximately 50F μm using 4000 grit SiC emery papers, and then discs with a diameter of 3 mm were punched out from the thinned slices. For further thinning, TEM foils were prepared by electrolytic thinning in a mixed solution of 10 ml HClO_4_ + 190 ml C_2_H_5_OH and cooled to −25 °C at 25 V using a twin jet polisher. Gatan DigitalMicrograph software (Gatan, Las Vegas, NV, USA) was employed for the TEM analysis. Meanwhile, to characterize the fracture behavior of the alloy, all the surface morphologies of the fractured tensile specimens were observed by SEM.

## 3. Results and Discussion

### 3.1. Microstructures

**Figure 4:**
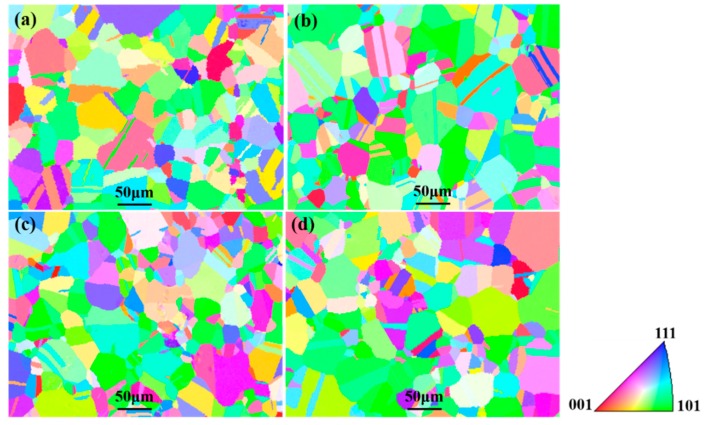
Inverse pole figures (IPFs) of specimens after various heat treatment processes. (**a**) Specimen A; (**b**) specimen B; (**c**) specimen C; (**d**) specimen D.

Figure 4 shows the inverse pole figures (IPFs) of specimens after various heat treatment processes. It was found that the orientation distribution of specimens was relatively random, indicating that heat treatment eliminated texture effects during sheet preparation. Additionally, IPFs displayed a fine uniform austenite grain structure. Furthermore, a certain amount of annealing twinning microstructures formed at the grain boundaries during the heat treatment process.

**Figure 5:**
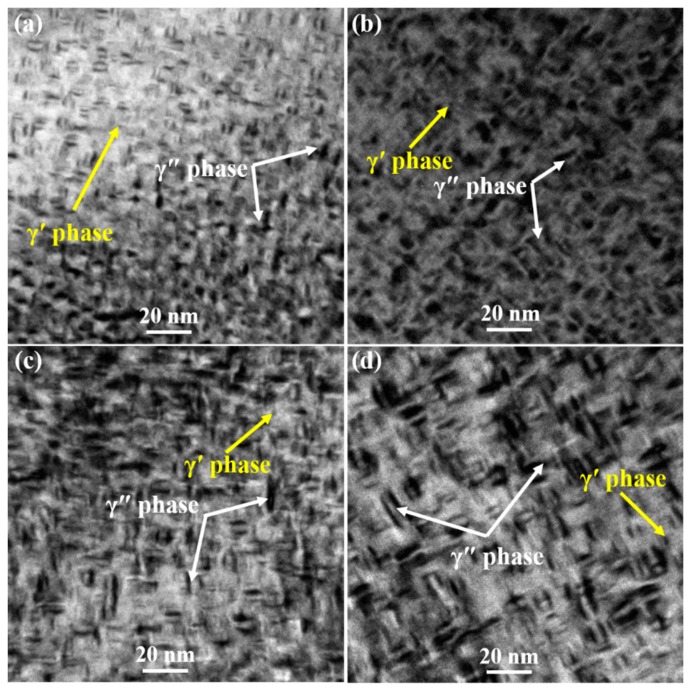
Morphologies of γ″ and γ′ phases. (**a**) Specimen A; (**b**) specimen B; (**c**) specimen C; (**d**) specimen D.

**Figure 6:**
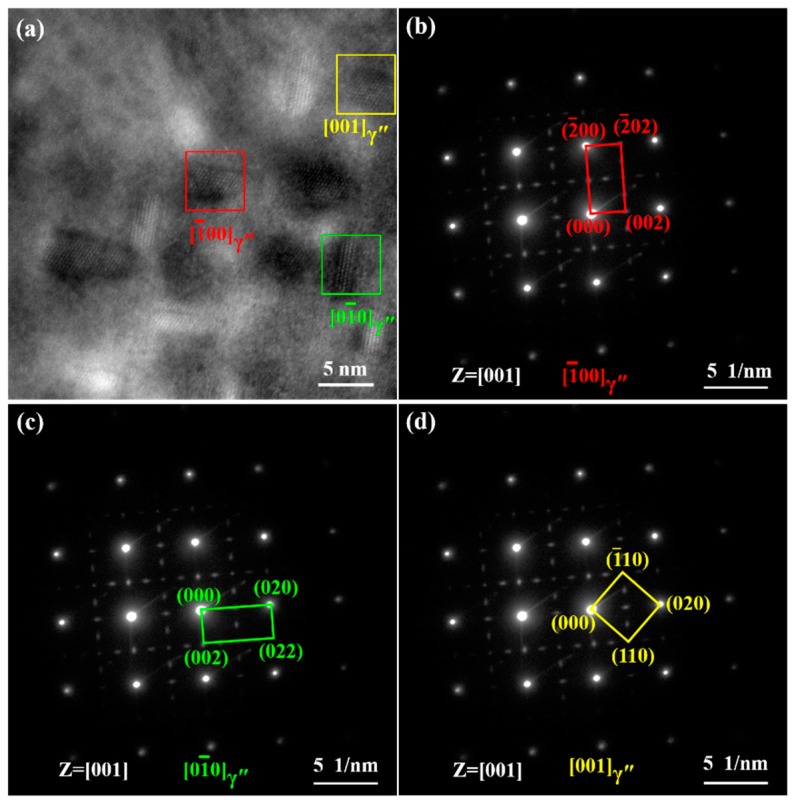
High-resolution TEM (HRTEM) image and selected area diffraction (SAD) patterns of γ″ phases in specimen A. (**a**) HRTEM image; (**b**) SAD pattern of ${\left[\bar{1}00\right]}_{\gamma^{\prime \prime }}$; (**c**) SAD pattern of ${\left[0\bar{1}0\right]}_{\gamma^{\prime \prime }}$; (**d**) SAD pattern of ${\left[001\right]}_{\gamma^{\prime \prime }}$.

**Figure 7:**
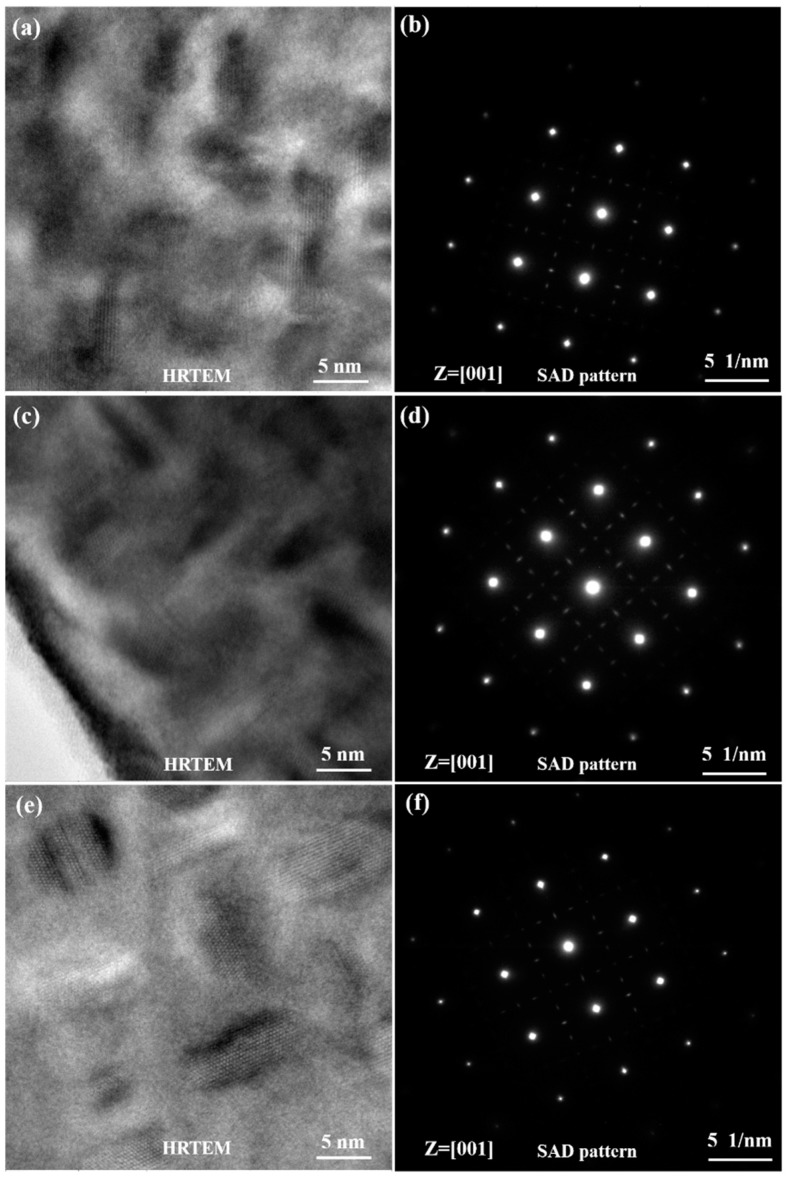
HRTEM images and SAD patterns of γ″ phases. (**a**,**b**) Specimen B; (**c**,**d**) specimen C; (**e**,**f**) specimen D.

Figure 5 shows the morphologies of γ″ and γ′ phases with various sizes and volume fractions. It was shown clearly in the TEM bright field (BF) images that the size and volume fraction of γ″ phases increased with the aging time. Three variants of the disc-shaped γ″ phases precipitated in the matrix γ phase for all specimen states. To clearly display three variants of the disc-shaped γ″ phases, high-resolution TEM (HRTEM) images and selected area diffraction (SAD) patterns are shown in Figure 6 and Figure 7. The γ″ phases precipitated corresponding to $\left[0\bar{1}0\right]$, $\left[\bar{1}00\right]$, and [001] orientations of the matrix γ phase. An accurate measurement of the volume fraction of the γ″ and γ′ phases was problematic [26] due to the very similar unit cell and chemical composition. Furthermore, the co-precipitated γ″ and γ′ phases resulted in complex γ′/γ″ co-precipitates [27,28]. Therefore, only the size of γ″ phases was measured by the TEM and HRTEM images analyses in this study. The average sizes of specimens A, B, C, and D were 4.4, 5.7, 7.2, and 9.0 nm, respectively.

### 3.2. Mechanical Properties

The 0.2% yield strength (YS), ultimate tensile strength (UTS), and elongation to fracture (EF) of Inconel 718 for various specimen states are listed in Table 1. The YS and UTS were sensitive to the precipitation of γ″ and γ′ phases. The YS and UTS increased with the increase in particle size and volume fraction of γ″ and γ′ phases. Generally, the EF could be used for the characterization of the plastic deformation capability of materials. It could be observed from Table 1 that an opposite tendency occurred for the EF, compared to that for the YS and UTS. The EF decreased with the increase in particle size and volume fraction of γ″ and γ′ phases. The coherent strengthening caused by γ″ phase and the order strengthening caused by γ′ phase were the main strengthening mechanisms for Inconel 718 superalloy [29]. Moreover, γ″ phase was the dominant strengthening phase and γ′ was the assistant strengthening phase. A stress field was easily induced by the mismatch in lattice parameters between the matrix γ phase and γ″ phase, which interacted with the stress field [30]. The precipitation of strengthening phases led to an increase in strengths at the expense of ductility [31].

### 3.3. Strain Evolution

**Figure 8:**
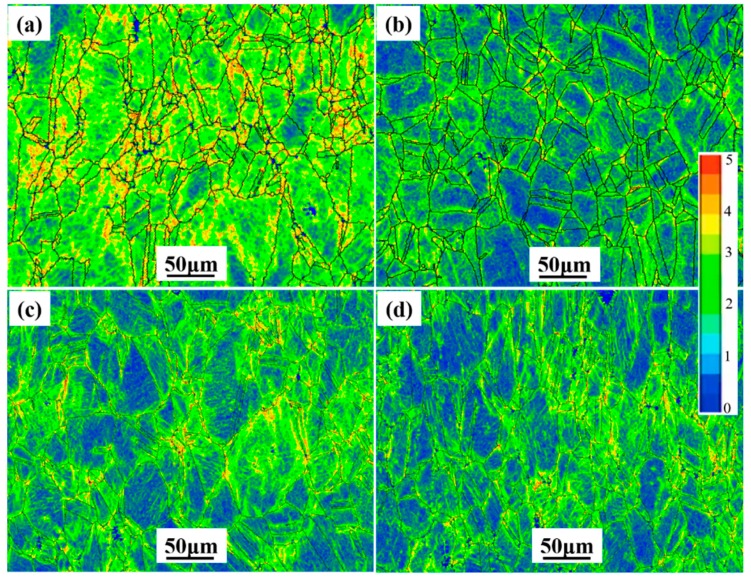
Kernel average misorientation (KAM) of the alloy measured for fractured tensile specimen surface. (**a**) Specimen A; (**b**) specimen B; (**c**) specimen C; (**d**) specimen D.

Two approaches can be used to essentially characterize plastic strain changes: (1) local misorientation in deformed materials and (2) the digital image correlation technique [32]. The kernel average misorientation (KAM) analysis of fractured tensile specimen surface is presented in Figure 8, where a large KAM value represents greater plastic deformation. It could be observed that the large KAM values were mainly located at the grain boundaries. This was because the grain boundary hindered the movement of dislocations, and then dislocations accumulated at the grain boundary during the plastic deformation. Furthermore, as the deformation continued, the plastic deformation evolved from the grain boundary to the inside of the grain. As shown in Figure 8a, the large plastic strain occurred in the inside of the grain of specimen A. This indicated that the grains produced obvious plastic deformation in specimen A after tensile deformation, more than that in specimens B, C, and D. This was closely attributed to the occurrence of γ″ and γ′ phases. The appropriate amount of γ″ and γ′ phases played a significant role in mechanical properties, while many γ″ and γ′ phases had a detrimental effect on the plasticity.

**Figure 9:**
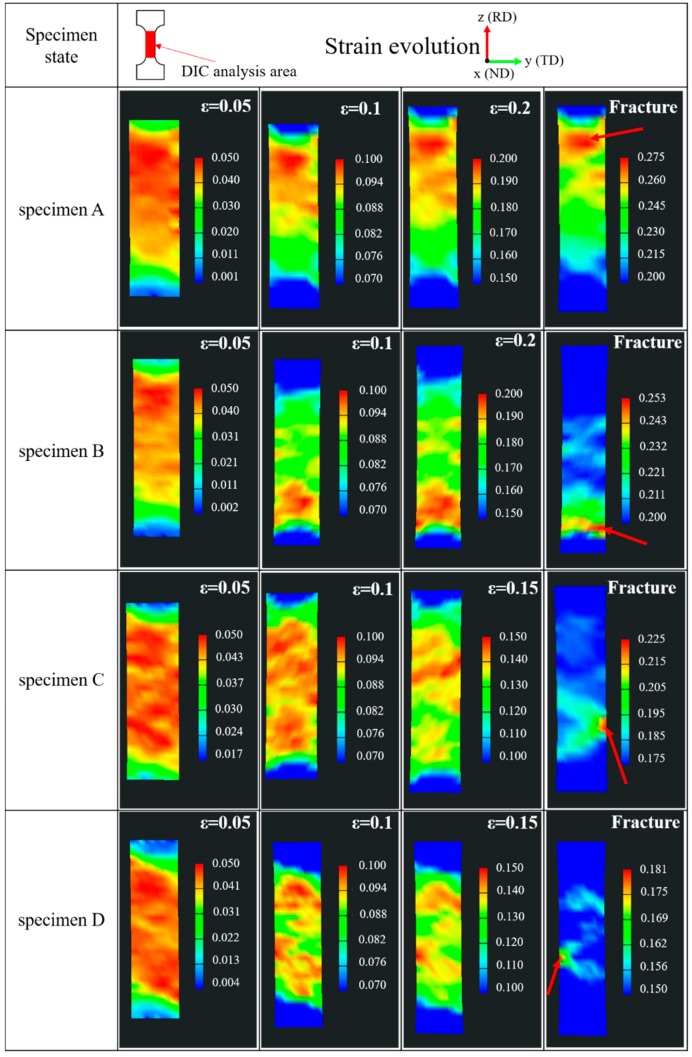
Real-time visualization of true strain evolution at the gauge length zone of specimens.

Figure 9 shows the real-time visualization of the true strain evolution at the gauge length zone of specimens during the tensile deformation. It could be observed that the true strain was relatively uniform at the beginning of plastic deformation. As the deformation continued, local strain began to appear and gradually increased until fracture occurred. The red arrows in Figure 9 indicate the eventual fracture location. The surface strain analysis showed that the deformation was more inhomogeneous along the RD with increasing aging time. It could be found that the strain concentration zones at the edge of the specimen were more obvious than those in the inside of the specimen. Additionally, as the plastic deformation continued, the degree of strain concentration at the edge of the specimen gradually increased. This may be because the water quenching effect resulted in an inhomogeneous temperature gradient between the edge and inside of the specimen, thereby causing a certain stress concentration at the edge of the specimen. Additionally, during uniaxial loading, the fixture of the test device may have introduced a fixed error, which led to inhomogeneous stress distribution on the cross-section. Also, the edge stress of the specimen was larger. Strain localization was mainly distributed in the center of the specimen for pure metal, and its inclination was about 45° with respect to the tensile direction [33]. However, the strain localization zones of the specimen with γ″ and γ′ phases scattered in different areas of the gauge length. The inclinations of some strain localization zones deviated from 45° with respect to the loading axis, which was closely related to the distribution of γ″ and γ′ phases in the specimens.

### 3.4. Fracture Mechanism

**Figure 10:**
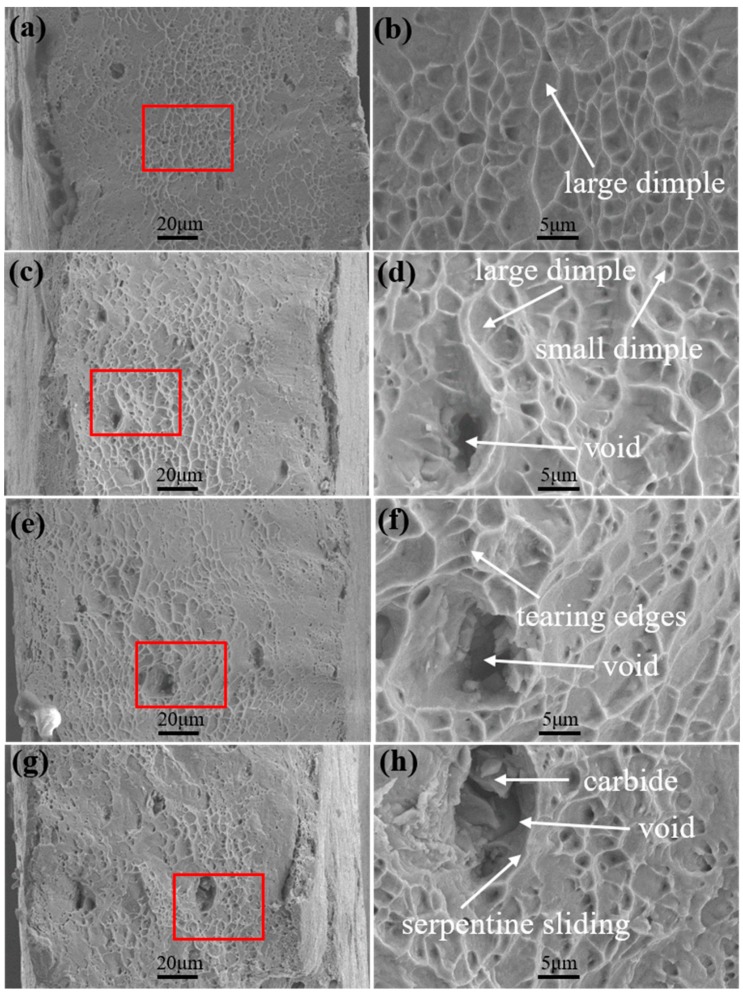
Fracture morphologies of specimens. (**a**,**b**) Specimen A; (**c**,**d**) specimen B; (**e**,**f**) specimen C; (**g**,**h**) specimen D.

The fracture morphologies of various specimens are displayed in Figure 10. The fracture surface was covered with dimples. The sizes of the dimples were distributed from 1 to 10 μm. Moreover, the size and number of the dimples in the vicinity of the fracture surface decreased with the increase in particle size and volume fraction of γ″ and γ′ phases. This implies that the superalloy with a small size and low volume fraction of γ″ and γ′ phases could tolerate higher damage before fracture. It could be concluded that the fracture mode was ductile fracture. Additionally, tearing edges (Figure 10f) and serpentine sliding occurred on the fracture surface (Figure 10h). Some carbides (NbC) were distributed in the voids. Generally, the carbides were harder than the matrix γ phase. However, the occurrence of γ″ and γ′ phases significantly strengthened the matrix γ phase, leading to the higher fracture limit of the matrix γ phase as compared to that of carbides. During the tensile deformation, γ″ and γ′ phases hindered the movement of dislocations, which resulted in the appearance of a dislocation pile-up phenomenon. Furthermore, the presence of carbides also hindered the slipping motion of mobile dislocations. These phenomena could result in an increased stress concentration. Once the stress concentration reached the fracture strength of carbides or the phase interfacial strength of carbides/matrix γ phase, the fracture of carbides occurred and voids nucleated around the carbides.

**Figure 11:**
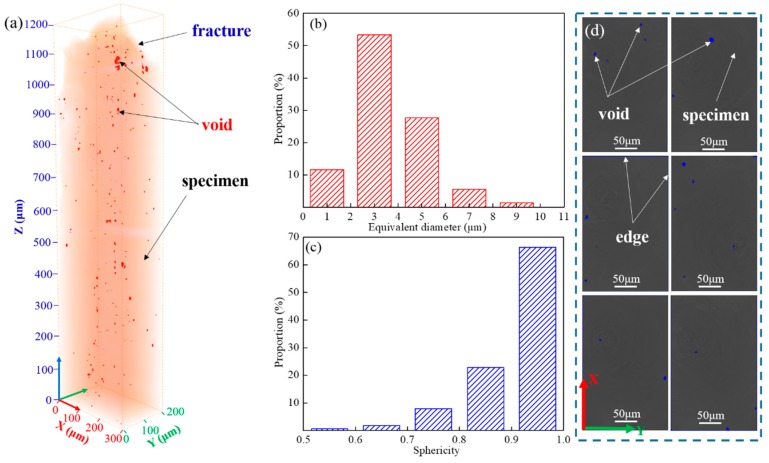
SRXT characterization results for fractured specimen B. (**a**) 3D reconstruction morphology; (**b**) distribution of void equivalent diameters; (**c**) distribution of void sphericity; (**d**) SRXT 2D slices of the fractured specimen cross-section.

Figure 11 shows the direct visualization of the spatial distribution of the void defects for fractured specimen B obtained by the SRXT technique. As shown in Figure 11a, different shaped red particles represent void defects inside the specimen. The characteristics of voids, such as the size and shape, could be obtained by SRXT and subsequent statistical analysis [34,35]. For a given particle, the measure of equivalent diameter computed the diameter of the spherical particle of the same volume. The distribution range of equivalent diameter values of voids mainly ranged from 2 to 6 μm (Figure 11b). For a given particle, the sphericity value represents the ratio of the surface area of a sphere with the same volume as the particle to the surface area of the particle [22]. The distribution range of the sphericity value of voids mainly ranged from 0.8 to 1 (Figure 11c), which showed that the shape of voids was mainly ellipsoidal. Figure 11d shows some SRXT 2D slices of the fractured specimen cross-section at a distance. They combined to make up a few cross-sections. The 2D slices of SRXT images were obtained by Avizo software. It could be observed that the proportion of voids distributed at the edge of the specimen was much higher than that of inside the specimen. Combined with the analysis results of DIC in Figure 8, this indicated that local strains at the edge of specimens corresponded to higher void densities. Moreover, local strains distributed at carbides or the phase interface of carbide/matrix γ phase also became the location of void initiation. The formation of voids in this superalloy was a significant process for void coalescence. When the voids were continuously extended and connected to each other in the tensile direction, the voids further formed large cracks due to the growth and coalescence of voids until the material finally broke.

## 4. Conclusions

In this study, various heat treatments were conducted to obtain tensile specimens with various γ″ and γ′ phases. The effect of γ″ and γ′ phases on RT tensile deformation and fracture behaviors of Inconel 718 was investigated. The main conclusions were as follows:

(1) Three variants of the disc-shaped γ″ phases precipitated corresponding to $\left[0\bar{1}0\right]$, $\left[\bar{1}00\right]$, and [001] orientations of the matrix γ phase. The size and volume fraction of γ″ and γ′ phases increased with the aging time.

(2) The strain concentration zones at the edge of specimens were more obvious than those in the inside of specimens. This was because the water quenching effect resulted in inhomogeneous temperature between the edge and inside of the specimen, thereby causing a certain stress concentration at the edge of the specimen. Additionally, the fixture of the test device may introduce a fixed error during uniaxial loading, leading to inhomogeneous stress distribution on the cross-section. In addition, the edge stress of the specimen was larger.

(3) SRXT characterization results for fractured specimens showed that the proportion of voids distributed at the edge of the specimen was much higher than that inside the specimen, which indicated that local strains at the edge of specimens corresponded to higher void densities.

(4) The size and number of dimples decreased with the increase in particle size and volume fraction of γ″ and γ′ phases. Local strains, which were distributed at carbides or the phase interface of carbide/matrix γ phase, became the location of void initiation. The fracture mechanism was ductile fracture.

## Figures and Tables

**Table 1:** Mechanical properties of Inconel 718.

Specimen State	Yield Strength (YS) (MPa)	Ultimate Tensile Strength (UTS) (MPa)	Elongation to Fracture (EF) (%)
Specimen A	756 ± 16	1090 ± 16	32.9 ± 2.0
Specimen B	902 ± 10	1222 ± 24	28.7 ± 1.6
Specimen C	945 ± 16	1284 ± 41	24.6 ± 1.5
Specimen D	1074 ± 36	1352 ± 30	21.0 ± 2.5
